# Predicting cancer-associated germline variations in proteins

**DOI:** 10.1186/1471-2164-13-S4-S8

**Published:** 2012-06-18

**Authors:** Pier Luigi Martelli, Piero Fariselli, Eva Balzani, Rita Casadio

**Affiliations:** 1 Biocomputing Group, *CIRI-Health Science and Technology/Department of Biology, via San Giacomo 9/2, Bologna, 40126, Italy; 2 Department of Computer Sciences, University of Bologna, via Mura Anteo Zamboni 7, Bologna, 40126, Italy

## Abstract

**Background:**

Various computational methods are presently available to classify whether a protein variation is disease-associated or not. However data derived from recent technological advancements make it feasible to extend the annotation of disease-associated variations in order to include specific phenotypes. Here we tackle the problem of distinguishing between genetic variations associated to cancer and variations associated to other genetic diseases.

**Results:**

We implement a new method based on Support Vector Machines that takes as input the protein variant and the protein function, as described by its associated Gene Ontology terms. Our approach succeeds in discriminating between germline variants that are likely to be cancer-associated from those that are related to other genetic disorders. The method performs with values of 90% accuracy and 0.61 Matthews correlation coefficient on a set comprising 6478 germline variations (16% are cancer-associated) in 592 proteins. The sensitivity and the specificity on the cancer class are 69% and 66%, respectively. Furthermore the method is capable of correctly excluding some 96% of 3392 somatic cancer-associated variations in 1983 proteins not included in the training/testing set.

**Conclusions:**

Here we prove feasible that a large set of cancer associated germline protein variations can be successfully discriminated from those associated to other genetic disorders. This is a step further in the process of protein variant annotation. Scoring largely improves when protein function as encoded by Gene Ontology terms is considered, corroborating the role of protein function as a key feature for a correct annotation of its variations.

## Background

The problem of annotating variations in proteins is particularly urgent given the high frequency of detection of non-synonymous Single Nucleotide Variations (SNVs) in humans thanks to the recent technological advancements in nucleotide sequencing. This direct approach allows the identification of common and also rare disease-associated germline variants that may play a role in susceptibility to different genetic disorders. Indeed a better knowledge of all the genes endowed with inherited variations will help case-control variation screening of human genetic diseases [1]. At present several available computational tools estimate with various scoring efficiencies whether a variation is or is not disease-associated, starting from the protein sequence and/or structure [2,3]. Recently the performance of prediction methods of variation pathogenicity on missense variants was assessed and two methods, SNPs&GO [4] and MutPred [5] scored with accuracies of 82% and 81%, respectively [6]. However the characterization of variations associated to specific phenotypes is still at its beginning. The vast majority of the methods [6] can classify the variations as disease associated or not with a likelihood of the prediction output without providing the type of associated pathogenicity. Alternatively, only few methods focus on variations that are known to be associated to specific disorders.

This is so particularly for cancer associated variations [7-9]. All the methods suited at predicting the cancer associated variations are based on the COSMIC dataset [10] containing both germline and somatic variations. The role of the individual somatic mutations in cancer pathogenesis and progression cannot be easily characterized and generally requires the application of computational filtering procedures. Karchin et al. [11] recently developed a method based both on features of the variation and of the protein at hand and on genomic information such as the conservation of genomic sequences among different species and the SNP density within exons as reported in HapMap [12]. By this their main result is the discrimination among driver and passenger mutations [11]. Our goal in this paper is different and complementary. Indeed we focus on germline variations and describe a newly implemented method that, taking as input the protein sequence, its function (as described with its associated GO-terms), and mutation type, well discriminates cancer-associated germline variations in proteins from those related to other genetic disorders. Our results support the notion that information on protein function helps in improving the performance of predictors suited at interpreting the phenotypic effects of protein variations.

## Methods

### GO-score

The GO-scores are computed with a formula previously defined [4]. For each protein this score is the sum of the log-odds associated to its GO annotations. The log-odd of each GO term is the logarithm of the ratio between its occurrence in “Cancer” class and in “Other diseases” class. We computed three different GO-scores according to the three different GO sub-ontologies (Molecular Function=F, Cellular Component=C, Biological Process=P). We always computed the GO-scores in cross-validation. This means that using the 10 protein subsets split by similarity (see Dataset section), we generated 30 different sets of GO-probabilities: 10 for each training set times 3 (the 3 GO-sub-ontologies). All the proteins in each test set were annotated by computing the 3 GO-scores of the protein terms using the GO-frequencies obtained in the corresponding training set.

### Implementing the method

We trained and tested Support Vector Machines (SVMs) with several kernel functions and here we report the results obtained with the best performing one: Radial Basis Functions (RBFs). We evaluated several types of input encoding based on protein features, evolutionary and functional information. The inputs encoded in the best performing predictors are:

- the variation type represented by a 20-valued vector where the wild type position is set to –1, the mutant residue position is set to 1 and all the other elements are set to 0 (indicated as “mut” in the Tables);

- the evolutionary information of the variation obtained by extracting the 4 columns that represent the wild-type and the mutant residues as reported by PSI-BLAST PSSM/PROFILE output generated using the -Q option (indicated as “E” in the Tables);

- a sequence profile window of dimension *x* centred into the mutated residue; the profile is encoded with 20*x elements vector (indicated as “Wx” in the Tables);

- the GO-scores encoded with 3 elements representing the log-odd scores of the three GO sub-ontologies (indicated as “GO” in the Tables).

### Measuring the performance

To asses the performance of the tested methods we counted in cross-validation the number of true positives (TP), true negatives (TN), false positive (FP), and false negatives (FN) with respect to either one of the two classes. We then computed the following indices:

Overall accuracy:(1)

Specificity:(2)

Sensitivity:(3)

Matthews correlation coefficient:(4)

## Results and discussion

### Dataset construction

For the training of newly implemented predictors, we collected from UniProtKB (http://www.uniprot.org) a first set of 6478 germline variations associated to diseases listed in OMIM (http://www.ncbi.nlm.nih.gov/omim). We only collected variations derived from SNVs and discarded all the variations annotated as “somatic” or “sporadic”. Furthermore we retained only variations for which a bibliographic reference reporting the association to a genetic disease is explicitly indicated in UniProtKB. Genetic disorders were grouped into two classes: “Cancer” and “Other diseases” on the basis of the OMIM disease descriptions (Table 1). The dataset consists of non-ambiguous variations: each mutated residue in a protein chain is univocally associated either to a cancer or other germline disease. Most proteins carry variations associated to a single disease class. Only 20 proteins contain different variations, some associated to cancer and some associated to other diseases. The dataset is available in Additional file 1. As to the functional annotation, 89%, 98%, and 96% of the proteins have an annotation for the Cellular Component, Molecular Function and Biological Process sub-ontologies, respectively. All proteins are endowed with at least one GO term annotation.

**Table 1:** Dataset of variations adopted for training/testing the method

Disease type	Number of proteins	Number of variations:	
		
		Cancer	Other diseases
Cancer	77	689	-

Other diseases	495	-	5026

Cancer and other diseases	20	358	405

Total	592	1047	5431

We adopted a cross-validation procedure to evaluate the predictors. We split the dataset into 10 cross-validation subsets. Sequences in one subset share <25% sequence identity with proteins in the complementary sets, according to an all-against-all BLAST search with E-value <0.001. For validating predictors, we also collected a second dataset of 3392 variations labelled “Somatic cancer” from UniProtKB present in 1983 proteins not included in the training set. This dataset does not include variants detected in cancer cell lines.

### Prediction of the disease type by protein similarity

One of the most popular method for protein sequence annotation is “transfer by similarity”. Here, the underlying basic idea is to transfer information from well characterized to poorly annotated proteins on the basis of their pairwise sequence similarity. We therefore quantified how relevant is sequence similarity on the discrimination of the two classes of germline variations (cancer versus other genetic diseases) and tested to which extent the similarity between two sequences biases the prediction of the disease type associated to their variations (Table 2). To this purpose, we first based our discrimination/prediction on the pairwise sequence identity as measured with BLAST. For each (query) protein *q* in our dataset, we ran a BLAST search against the dataset after setting a very high E-value threshold (E-value=100). Protein hits are then considered according to BLAST sorting (from the lowest to the highest E-values) and for each protein *q* in the data set only the best-hit protein is retained from the BLAST list. This procedure allows the selection of the top scoring proteins (if any) as candidates for “transferring” the disease annotation to the query protein. Transfer was done by applying a majority rule: when the top scoring protein *p* had a number of cancer associated variations higher than that associated to “other diseases”, all the variations of the query protein *q* were labelled/predicted as cancer associated; otherwise all the variations of the query protein *q* were labelled/predicted as “other diseases”. Scoring indexes were evaluated accordingly. In Table 2, the performance of the *disease prediction by similarity* is listed at increasing threshold cut-off of the percentage identity between the query and the BLAST best-hit. Interestingly our data prove that when the sequence identity threshold cut-off is 60%, 99% of all the proteins in the data set can be disease annotated (the corresponding BLAST best-hit is 60% identical or less) with an overall accuracy of 0.76 and a Matthews correlation coefficient of 0.26. The low MCC value is indicative of the highly unbalanced data set at hand. At increasing values of sequence identity both accuracy and MCC values slightly increase, while at decreasing identity values the performance decreases. When sequence identity threshold is 30%, the MCC value is close to random and only 10% of the proteins in the data set have corresponding hits. Summing up, this analysis performed on the available and unbalanced data set presently available (Table 1), indicates that when proteins are highly similar their variations may inherit an annotation for the disease type based on the procedure of transfer by similarity. However below this identity threshold annotation requires other approaches as described in the following sections.

**Table 2:** Prediction of the disease type by protein similarity

% Id	% prot	Sp(C)	Sp (N)	Sn (C)	Sn (N)	MCC	Q2
≤30	10	0.13	0.94	0.25	0.87	0.09	0.83

≤40	75	0.26	0.85	0.29	0.83	0.11	0.74

≤50	95	0.28	0.87	0.41	0.80	0.18	0.73

≤60	99	0.34	0.88	0.47	0.82	0.26	0.76

≤70	100	0.35	0.89	0.46	0.83	0.26	0.77

≤80	100	0.36	0.89	0.48	0.83	0.29	0.78

≤90	100	0.36	0.89	0.48	0.83	0.29	0.78

%prot= percentage of proteins that can be annotated with a given similarity threshold cut-off. %Id= Threshold cut-off of the sequence identity of the best hit retrieved upon a BLAST search in our dataset. For a definition of classes and scoring indexes see section: Measuring the performance.

### Prediction of the disease type by protein function

The unbalanced distribution of the available data set reported in Table 1 shows that the majority of the proteins has variations associated to a single disease type (in this binary view, familial cancer and non cancer). This can be explained assuming that the protein itself and/or its biological role can carry a significant amount of information for the task at hand. We then evaluated the role of functional information, a property of the whole protein, in the prediction of the disease type. To label protein function we took advantage of the Gene Ontology (GO) annotation present in UniProtKB. Labelling of protein variants in the lack of any other source of information was done as in the previous experiment (see previous section), assigning all the variants in the same sequence to the same disease type.

We then applied a 10-fold cross-validation to compute the GO-scores on the 10 different subsets including the proteins of our data set (as described in the Methods section). By this, each prediction based on the protein GO-terms was done without including the GO-terms of the protein to be classified (nor all the other GO-terms of the proteins in the same test subset).

In Table 3 we list the classification performance of the variants as germline cancer-associated or other familial disease-associated based on cross validated GO-scores. With this procedure, depending on the sub-ontology, not all the variations could be predicted (“% var” column in Table 3). Furthermore, due to specific information, variations in the same protein carrying different labels cannot be discriminated (the number of proteins with variants labelled differently and presently available is 20, corresponding to 3.4% of the whole data set, as shown in Table 1). The best performing sub-ontology is “Biological Process” (P in Table 3) scoring with 89% accuracy on 96% of the variations. When the average score of the three sub-ontologies is considered, the total number of variations can be predicted (last row of Table 3); however scoring values decrease, suggesting that the average GO-score is not sufficient to optimise classification. Since the GO-scores discriminate quite well between the two disease classes, we analysed the GO-terms that contribute the most to the prediction process.

**Table 3:** Prediction of the disease type by protein function

GO sub-ontology	% var	Q	MCC	Sp(C)	Sn(C)	Sp(O)	Sn(O)
C	89	0.76	0.3	0.58	0.34	0.79	0.91

F	98	0.77	0.45	0.83	0.39	0.75	0.96

P	96	0.89	0.63	0.79	0.62	0.9	0.96

CFP	100	0.75	0.52	0.40	0.97	0.99	0.71

GO sub ontology: C=cellular component, F=molecular function, P=biological process. % var= percentage of predicted variations. Predicted classes: C= Cancer; O= Other genetic diseases. For a definition of classes and scoring indexes see section: Measuring the performance.

From the analysis described above we can conclude that the most discriminative GO-terms in the Biological Process sub-ontology are related to DNA-repair, microtubule regulation, and catabolic processes (Table 4). Variations affecting proteins related to DNA-repair increase the rate of replication errors, while defects in microtubule regulation are known to affect the regular course of cell-cycle, including mitosis [13]. In the case of impairment of catabolic processes, alternative and potentially oncogenic pathways (e.g. are hypoxia pathway) are often adopted by the cell [14]. When considering the Molecular Function sub-ontology, most discriminative terms relate to DNA repair and kinase activity that are known to be implicated in signalling pathways regulating the cell cycle (Table 4). The most discriminative terms related to the Cellular Component sub-ontology refers to DNA-repair complexes MutSalpha and MutSbeta, to lysosome and lytic vacuoles, involved in apoptosis and tumour suppression processes [15], and to catenin complex, a key regulator of the Wnt signalling pathway whose alterations are associated to carcinogenesis [16]. Overall, our findings are in agreement with the notion that variations affecting proteins involved in these processes/functions can be related to oncogenesis and cancer progression.

**Table 4:** Most discriminative GO annotations

GO-term	Description
***Cellular Component* (*C*)**	

GO:0032301	MutSalpha complex

GO:0032300	Mismatch repair complex

GO:0032302	MutSbeta complex

GO:0005773	Vacuole

GO:0005764	Lysosome

GO:0000323	Lytic vacuole

GO:0030877	Beta-catenin destruction complex

GO:0016328	Lateral plasma membrane

GO:0034747	Axin-APC-beta-catenin-GSK3B complex

***Molecular Function* (*F*)**	

GO:0030983	Mismatched DNA binding

GO:0032137	Guanine/thymine mispair binding

GO:0032134	Mispaired DNA binding

GO:0030291	Protein serine/threonine kinase inhibitor activity

GO:0016538	Cyclin-dependent protein kinase regulator activity

GO:0004861	Cyclin-dependent protein kinase inhibitor activity

GO:0019887	Protein kinase regulator activity

GO:0019207	Kinase regulator activity

GO:0005099	Ras GTPase activator activity

***Biological Process* (*P*)**	

GO:0006298	Mismatch repair

GO:0044271	Cellular nitrogen compound biosynthetic process

GO:0006301	Postreplication repair

GO:0046395	Carboxylic acid catabolic process

GO:0016054	Organic acid catabolic process

GO:0009310	Amine catabolic process

GO:0070507	Regulation of microtubule cytoskeleton organization

GO:0032886	Regulation of microtubule-based process

GO:0009063	Cellular amino acid catabolic process.

### Prediction of the disease type with a SVM-based method

In order to improve the method performance and including also the possibility of predicting variations associated to different disease types in the same protein, we implemented different SVM based predictors taking advantage of the results described above. Practically we implemented different SVM based predictors with and without protein GO-scores. The results (Table 5) highlight that the performances are significantly lower when the GO-scores are not included in the input encoding (first two rows). Noticeably the SVM-based predictor, encoding only the three GO-scores, performs significantly higher than the simple average of the scores (compare last row of Table 3 with the third row of Table 5). The best performing predictor of Table 5 is a SVM based method that adds to the GO-scores also the variation type (last row of Table 5). Noticeably with this last implementation it is possible to assign classifications to variations labelled differently in the same protein sequence (endowed with the same GO-scores). Although the set of proteins carrying differently labelled variations (germline “cancer” and germline “other diseases”) is small (20 proteins, Table 1), a total amount of 358 germline “cancer” and 405 germline “other diseases” variations can be predicted with the SVM based method. On this set the efficacy of the GO terms vanishes. However the predictor scores with accuracy and MCC equal to 70% and 0.4, respectively. These values are lower than those obtained on the whole dataset (90% and 0.61) but still significantly different from random. Overall our results strengthen the notion that functional annotation is needed for the optimal prediction and that the integration of the variation type in the input encoding can help in difficult cases where both cancer and other diseases are associated to different variations in the same sequence.

**Table 5:** Prediction of the disease type with a SVM-based method

Encoding	Q	MCC	Sp(C)	Sn(C)	Sp(O)	Sn(O)
mut_E_W1	0.59	0.12	0.21	0.55	0.88	0.60

mut_E_W5	0.64	0.17	0.24	0.55	0.88	0.66

OnlyGO	0.89	0.60	0.7	0.63	0.93	0.95

mut_GO_E	0.89	0.60	0.68	0.65	0.93	0.94

mut_GO_E_W1	0.89	0.58	0.68	0.64	0.93	0.94

mut_GO_E_W5	0.89	0.58	0.68	0.64	0.93	0.94

mut_GO	0.90	0.61	0.69	0.66	0.93	0.94

mut= is a 20 elements vector that encodes for the variation type; Wx= a input sequence window of dimension x centered into the variation; E= the evolutionary information on the variation obtained by extracting the 4 columns that represent the wild-type and the mutant residues as reported by PSI-BLAST PSSM/PROFILE output (-Q option); GO= the 3 GO scores. For a definition of classes and scoring indexes see section: Measuring the performance.

We also predicted with the most accurate SVM of Table 5 a set of 3392 variations associated to “Somatic cancer” in 1983 proteins not included in the training/testing set. On this set the predictor correctly discharges 96% of the variations with a false positive rate of only 4% mispredicted cases. This indicates that our method can indeed quite accurately discriminate between cancer related germline variations and somatic ones.

### Integration of the SVM-based method with SNPs&GO

Our new implementation classifies variations that are disease related. This implies that it can be used in experimental set-ups or in pipelines in cascade with other more general predictors discriminating among disease related and neutral variations. In this respect we devised an experiment: evaluation in a cascade with our SNPs&GO [4]. We predicted with SNPs&GO variations of the proteins in our dataset taking care of removing the sequence identity with those of the SNPs&GO training set. To this aim, we used the 10 different cross-validated SNPs&GO models and for each target sequence to be predicted we adopted only the model trained on a set that does not contain sequences similar to the target. SNPs&GO predicts as disease related 70% of the variations of our dataset. This result was expected, since SNPs&GO was tuned to have a very low rate of false positives (variations predicted as disease but observed as neutral) at expenses of the coverage (some disease related variations are discharged as neutral). However, when SNPs&GO is used in combination with our best SVM-based method (mut_GO in Table 5) the combined accuracy in discriminating between germline cancer and non cancer associated variations is higher than that of the single method alone (Table 6). This suggests that even if only 70% of the variations were correctly retained by SNPs&GO, the missed variations are probably the less discriminative between germline variations associated to cancer and other diseases.

**Table 6:** Cross-validation performance of a SVM-based predictor in cascade with SNPs&GO

Method	Q	MCC	Sp(C)	Sn(C)	Sp(O)	Sn(O)
mut_GO+(SNP&GO)	0.92	0.67	0.79	0.65	0.94	0.97

mut_GO	0.90	0.61	0.69	0.66	0.93	0.94

For a definition of classes and scoring indexes see section: Measuring the performance.

## Conclusions

Overall our work aims at filling the gap between predictors classifying variations as disease-associated or not and association studies among genotypes and phenotypes [17]. In this paper we focus on discriminating cancer germline from other variations associated to genetic diseases. Our results indicate that protein function, when integrated with the variation information in a SVM based method, is a key feature for a correct classification.

Furthermore, when the method is applied to cancer-somatic variations it predicts most of them as non associated to cancer germline variations. Our predictor can therefore be applied to prioritize germline variations in proteomes of cancer cells.

## Competing interests

The authors declare that they have no competing interests.

## Authors' contributions

PF, PLM and RC conceived the paper. EB and PLM built the annotated dataset. EB, PF and PLM performed predictions. All the authors analyzed the results and co-authored the paper.

## Supplementary Material

Additional file 1 Data set Click here for file
